# Quality Improvement Opportunities in Caring for Patients with Nonepileptic Seizures

**DOI:** 10.1155/2014/201575

**Published:** 2014-09-09

**Authors:** Jasper J. Chen, Devendra S. Thakur, Krzysztof A. Bujarski, Barbara C. Jobst, Erik J. Kobylarz, Vijay M. Thadani

**Affiliations:** ^1^Behavioral Health Services, Cheyenne Regional Medical Center, Cheyenne, WY 82001, USA; ^2^Department of Psychiatry, Geisel School of Medicine at Dartmouth College, Dartmouth-Hitchcock Medical Center, Lebanon, NH 03756, USA; ^3^Department of Neurology, Dartmouth-Hitchcock Medical Center, Lebanon, NH 03756, USA

## Abstract

*Background*. Patients with nonepileptic seizures (NES) are challenging to treat for myriad reasons. Often patients may be misdiagnosed with having epilepsy and then may suffer unintended consequences of treatment side effects with antiepileptic medication. In addition, patients may be maligned by health care providers due to a lack of ownership by both psychiatrists and neurologists and a dearth of dedicated professionals who are able to effectively treat and reduce severity and frequency of symptoms. *Aims of Case Report*. Many psychiatrists and neurologists are unaware of the extent of the barriers to care faced by patients with NES (PWNES) and the degree of perception of maltreatment or lack of therapeutic alliance at various stages of their care, including medical workup, video-EEG monitoring, and follow-up plans. We present the case of a patient with NES who experienced numerous barriers as well as incoordination to her care despite being offered a breadth of resources and discuss the quality improvement opportunities that may exist to improve care of patients with NES. *Conclusion*. No known literature has documented the extensive barriers to care of PWNES in parallel to quality improvement opportunities for improving their care. We endeavor to contribute to the overall formulation and development of a clinical care pathway for PWNES.

## 1. Background

Nonepileptic seizures (NES) are paroxysmal episodes of altered behavior that may resemble epileptic seizures but lack electroencephalographic (EEG) epileptic changes and the association to central nervous system dysfunction [1]. The prevalence of NES has been estimated to range from 1/50,000 to 1/3,000 [2]. Patients with NES (PWNES) may account for upwards of 1/3rd of the population admitted to epilepsy monitoring units of tertiary academic medical centers for intractable seizures [3]. It has also been reported that approximately 11% of PWNES have epileptic seizures [4].

Myriad studies have found a high degree of prevalence over the lifetime of multiple psychiatric disorders among PWNES [5]. In fact, simultaneous presence of two or more psychiatric diagnoses may exist nearly in three-quarters of PWNES [6]. The literature has suggested that 91% of PWNES have dissociative disorders, 64% have mood disorders, 49% have posttraumatic stress disorder (PTSD), 47% have anxiety disorders, 42% have substance abuse disorders, and 30% to 50% have personality disorders, the most frequent of which are borderline personality and histrionic disorders [6].

While previous reports have delineated the challenges generally and inherently involved in the differential diagnosis between NES and epilepsy [7], we are unaware of any attempts to specifically identify quality improvement opportunities to enhance the overall treatment approach to PWNES via a well-defined clinical care pathway. Our case report therefore illustrates how a clinical pathway may expedite and optimize the care plan for a PWNES.

## 2. Case Report

### 2.1. Early Clinical History and Treatments

We present the case of a 22-year-old right-handed female patient referred originally to our neurology department by an outside primary neurologist for seizures. Her early development was delayed when she graduated from high school but required special education classes; she attended some college but did not receive a degree. She was raised by her mother, on whom she relies for support, as her father passed away while she was young. Her family history was negative for epilepsy. She endorsed a positive history of sexual and traumatic abuse. She eventually received medical disability for anxiety and Tourette's syndrome.

Specifically, she started to exhibit tics and anxiety at the age of 5 years. Her tics consisted of grunts, vocalization, and hand-flapping. She also exhibited obsessive-compulsive traits, for which she was treated effectively with pimozide, but which eventually had to be discontinued due to very intolerable side effects, including galactorrhea. Subsequently, she was treated with clomipramine, although some symptoms including coughing and other vocal tics persisted, such as occasional extremity twitching.

The patient was evaluated at our epilepsy monitoring unit (EMU) initially during 2010 for video-EEG (VEEG) monitoring for headaches and paresthesias. During this EMU admission, her brain MRI was normal. Her EEG revealed some scattered, sharp activity with a posterior predominance that was not clearly epileptic.

### 2.2. Evaluation of New Symptoms

In 2013, the patient began to experience staring spells, which coincided with a particularly difficult time in college during which she was sexually harassed. Thrice weekly, she was staring off into space and becoming unresponsive for about a minute. Along with these episodes, the patient was also observed to have convulsive seizures, some of which were triggered by positional changes and orthostatic symptoms. The patient's mother reported that during these latter episodes she would become unresponsive and exhibit varying degrees of convulsive bilateral seizure activity lasting up to a minute.

The patient had poor recollection for these events and there was no tongue-biting or incontinence. The patient also described a third type of nocturnal spell during which she would experience nightmares and then would develop a sensation of choking or drowning upon awakening.

Outpatient evaluation at the time of the initial neurology referral included a normal brain MRI scan and an EEG showing scattered generalized sharp theta activity during drowsiness which was not clearly abnormal. Holter monitoring captured some dizziness spells, including light-headedness and palpitations, without concurrent ECG abnormalities. The patient also reported that she had not been previously treated with any antiepileptic medication.

For the patient's anxiety and Tourette syndrome, she was referred to outpatient psychiatry for further evaluation and management, which consisted of a retrial of clomipramine, as well as augmentation strategies with hydroxyzine and quetiapine. These efforts resulted in partial improvement. However, after two months, the patient felt that clomipramine may have exacerbated her spells, and she discontinued this medication. A gabapentin trial was then prescribed for mood stabilization, and the patient's anxiety improved. The patient was also encouraged to reengage in cognitive behavioral therapy, which had previously helped the patient as a teenager.

Subsequently, the patient developed new spells which, per the patient's and her mother's report, consisted of tongue-biting episodes, urinary incontinence, and postictal confusion lasting for 30 minutes. She described having up to half dozen events per day and often did not return to baseline afterwards. She would often experience a premonition followed by sitting or lying down. These events typically lasted for less than one minute.

Due to failure of increased gabapentin from 600 mg TID to 900 mg TID by the patient's primary outpatient neurologist, repeat elective VEEG monitoring for new spell characterization was planned. The patient's primary outpatient neurologist had determined to try to capture any episodes possibly epileptic to consider dual diagnosis of NES and epilepsy. However, the results of this repeat VEEG monitoring proved that the new spells were clearly nonepileptic.

These findings were discussed at length with the patient and her mother, who from the beginning described herself as “the patient's primary advocate because nobody else will advocate for her.” Upon repeated and variable questioning and discussion, the patient vehemently denied that her spells could be exacerbated by any psychosocial stressors. Furthermore, both the patient and her mother endorsed that her tic disorder and obsessive-compulsive disorder had actually improved as compared to several months prior.

### 2.3. Evaluation of Psychiatric Comorbidities and Difficulties with Care Coordination

Of particular psychodynamic interest, the patient had undergone hysterectomy at an OSH locally, and the patient's primary outpatient neurologist knew in advance that she was going to have the hysterectomy. However, she and her mother were fully committed, and the OSH ethics board had signed off on it, so the primary outpatient neurologist felt that there was nothing to be done to stop this process. The hysterectomy occurred approximately 4–6 weeks prior to her 2nd EMU admission.

Her reasoning was that she did not want to “pass on my genes to another poor individual.” The OSH had consulted their ethics review board prior to performing the procedure. When the epilepsy team had learned of the hysterectomy, the inpatient psychiatry consultation and liaison services were immediately consulted over their “concern that the patient could have had a hysterical reaction and was dissociating in response to having her womanhood violated.”

The psychiatric consultation service was initially taken aback by the consultation request since the patient's primary outpatient psychiatrist, a senior psychiatry resident, was rotating on the epilepsy team directly caring for the patient. However, the chief resident and epilepsy attending on service conferred with the patient's primary outpatient neurologist to determine that neurology had nothing further to offer the patient. A plan was devised for the patient to be transferred to the psychiatry inpatient unit for further evaluation of dissociative episodes suggestive of PTSD symptoms—hypothesized following hysterectomy—or to devise a more tenable outpatient treatment plan.

Psychiatry consultation found that the patient was primarily anxious regarding her physical symptoms because there appeared to be no known identifiable psychosocial stressors. In fact, she had recently started a new job as a waitress which pleased her and earned her new friends. During the interview, the patient was quite focused on her physical symptoms, including her tachycardia sensation, which appeared to precipitate or exacerbate her anxiety.

The patient also demonstrated low self-esteem as she frequently talked about being “ridden with disability.” She stated, “I am not fit to have children because of my disability and do not want to pass on my conditions to future generations,” thus causing the patient to decide to undergo a hysterectomy. Evaluation by psychiatry revealed the patient to have dependent traits, mostly on her mother, and recommended further consideration of longitudinal psychotherapy. Notably, the patient had been receiving on-going psychiatric care in the setting of a year-long pilot quality improvement intervention consisting of a psychiatry clinic embedded within the epilepsy clinic [7].

At the patient's follow-up appointment after discharge from her 2nd EMU admission, the patient and her mother shared with the primary outpatient psychiatrist the fact that they were told by the primary outpatient neurologist first that “the underlying problem is possible brain damage around the time of birth and mental stressors can make all these problems worse, but do not cause them directly” but that “later on he changed his mind, so we are very confused.” The patient and her mother expressed uncertainty of their primary neurologist's ability to actually help them and that they had been advised to seek psychiatric care as opposed to neurologic care.

## 3. Discussion

We are unaware of any reports specifically investigating the barriers to care for PWNES and document the extent of incoordination of care for patients receiving treatment even within a single institution. The literature regarding clinical pathways may be relevant to our desire to better standardize the care for PWNES. Briefly, clinical pathways—dedicated care plans for target populations—are used to translate guidelines into local practices [8]. The outcomes from the majority of clinical care pathways have resulted in significantly lower cost and other surrogate measures in terms of hospitalization costs and charges or insurance points for pathway groups [8].

Therefore, clinical care pathways are associated with a more efficient use of resources and efficiency of care [8]. Thus far, there have been insufficient efforts to develop quality improvement interventions to ameliorate barriers to care for PWNES. However, cooperation between neurologists and psychiatrists in a multidisciplinary approach has been suggested as a requirement to provide an appropriate medical home [9] for a “borderland disorder” [10] such as NES.

On the basis of our presented patient's experience, we therefore propose the creation of a clinical care pathway for PWNES:

1. opening channels of regular communication, including or leading up to the creation of a joint NES clinic as a collaborative venture between departments of psychiatry and neurology to avoid uncoordinated care for PWNES;
2. early VEEG monitoring for patients suspected to have NES on clinical history as well as consideration of repeat VEEG monitoring as appropriate and within reasonable limits; awareness that it may take more than one EMU admission to more reliably and confidently diagnose NES as was in the case in our presented report;
3. coordinated appointments between patients' primary outpatient neurologists and psychiatrists to optimize provider role-specification and minimize patients' splitting their providers;
4. dedicated colocated psychiatrist or behavioral health clinician including licensed independent clinical social workers (LICSW) within the epilepsy clinic who has either special training (e.g., dual residency training in neurology and psychiatry or psychosomatic fellowship), developed clinical acumen, or a personal interest in caring for PWNES;
5. regular rounding by the colocated psychiatrist with the primary epilepsy team to proactively consult on patients undergoing EMU admission with likely/suspected NES due to lack of epileptic activity on VEEG; in this manner psychiatric care would commence prior to discharge from the EMU;
6. building a curriculum to offer group therapy sessions for PWNES for group validation, sharing of frustrations, and normalization of their experiences; there may be a need for a therapeutic approach that ultimately would be very similar to Marsha Linehan's dialectical behavioral therapy (DBT) [11] in terms of emphasizing empathic validation of symptoms and fostering awareness and insights of possible relationships between psychosocial stressors and the physical and emotional creation of treatable symptomology;
7. offering structural family therapy to patients and their families as appropriate (as may have been the case for understanding the dynamics between this patient and her mother);
8. offering support group sessions for families of PWNES;
9. offering vocational rehabilitation services to PWNES with the hope that meaningful work, either for remuneration or on a voluntary basis, may help with providing more purpose and meaning in lives of PWNES;
10. Please refer to Table 1 for patient-, provider-, and system-related factors that may be more or less amenable for referral of PWNES to psychiatric or psychology providers.

## 4. Limitations

The implementation of all elements of the aforementioned clinical pathway for PWNES would be more difficult in more remote areas or in areas limited to fewer specialists. A potential serious limitation to our proposed clinical pathway is that, as both neurology and psychiatry services are often quite busy, simultaneous rounds and appointments may not be feasible in many centers. Opportunities for even improved communication in terms of coordinating care between local psychiatrists and neurologists would enable PWNES to fare better. Having an opportunity for PWNES to see both a psychiatrist and a neurologist either simultaneously or having appointments within a short amount of time of each other would not only foster collaboration but also enhance clinical decision making. When well coordinated, the absolute necessity for a joint clinic could be obviated, although the latter would perhaps be more optimal in a larger, academic medical center setting.

Therefore, given the reality of clinical constraints, education of patients and families is imperative in optimally caring for patients with nonepileptic seizures, in addition to group support sessions. It may be equally important to help patients understand what nonepileptic seizures are as what they are not. We believe that educating psychiatrists and neurologists alike to specify roles in caring for patients with NES is a very important first step towards eventual implementation of all or parts of the proposed clinical pathway.

It is also prudent to discuss the neurological view of psychogenic seizures and their perceived role in care, as this is potentially a very significant treatment barrier. Whitehead et al. [12] found that neurologists—much more often than their patients—believe in the mind-body duality of psychosomatic symptomology relating to the etiology of seizure disorder. However, distinguishing purely “organic” versus “psychological” bases for seizures and NES, respectively, is artificial and can potentially invalidate the subjectively experienced symptoms of patients with NES.

In fact, research shows significant overlap in patients with epilepsy and patients with NES in that many patients with NES are eventually diagnosed with comorbid epilepsy and many patients with epilepsy are eventually diagnosed with comorbid NES [7]. We must additionally always remember that, as they have a conversion disorder, patients with NES do not have conscious control of either producing or voluntarily producing their symptoms. Often clinicians—albeit unconsciously—lose sight of this most important point.

Lastly, regarding the care of our presented patient, while a joint clinic may not have necessarily resulted in better coordinated care, latency to receiving psychiatric care might have been decreased. At the very least, having clearly defined standardized operating procedures may better allow patients to receive psychiatric consultation when individuals are identified as having nonepileptic seizures. Principally, the argument for optimally having a joint clinic or even a shared appointment is to increase communication between neurologic and psychiatric providers, minimize any potential splitting of providers by patients, and prevent providers from shirking responsibility by claiming the other provider to be the primary care provider.

## 5. Conclusion

The dearth of available, well-trained, and experienced providers calls for the creation of a clinical pathway specifically for PWNES to better coordinate and consolidate their care. Some have argued for more serious conversations with the Centers for Medicare and Medicaid Services (CMS)—similar to efforts in the field of dementia—to discuss program development to deliver patient-centric, comprehensive, and multidisciplinary healthcare to PWNES [10]. This clinical case illustrates the benefits of implementing a clinical pathway devoted to the care of PWNES. We would also encourage greater availability of major funding sources to promote more widespread interest in treating somatoform and conversion disorders and to entice psychiatrists to become more actively engaged in study design processes for PWNES [13].

## Figures and Tables

**Table 1:** Patient, provider, and system factors contributing to care for patients with nonepileptic seizures and characteristics triggering psychiatric or psychological referral.

Factors	Less suited towards referral	More suited towards referral
Patient and family factors	Belief in stigmatization of psychiatric care and that it may not be helpful	Acceptance that psychiatric referral can be helpful and is not stigmatizing
	Belief in external locus of control of symptoms	Belief in internal locus of control of symptoms
	Fair to good insight and awareness of psychosocial stressors	Poor to fair insight and awareness of psychosocial stressors
	More emotionally supportive and more physically present family and or friends	Less emotionally supportive and less physically present family and or friends

Provider factors		
Neurologist (referring physician) factors	Comfortable and/or has sufficient time and energy to manage psychiatric comorbidities	Less comfortable and has insufficient time to manage psychiatric comorbidities
Psychiatrist (receiving physician) factors	Not as interested in care of patients with neurological disorders or not believing that psychiatric/psychological interventions may be helpful	Comfortable with and has predilection to manage affectively challenging patients

System factors	Lack of availability of psychiatrists and psychologists locally	Availability of psychiatrists and psychologists as well as opportunities to build “shared appointments” with neurologists
	Inability of neurologists and psychiatrists to dedicate sufficient time to care coordination for patients with NES	Ability of neurologists and psychiatrists to communicate effectively and have regular meetings and or open channels of communication regarding care coordination for patients with NES
